# Making Sweat Measurable: Induction, Sampling, and Refreshment in Wearable Biofluid Sensing

**DOI:** 10.1002/advs.75684

**Published:** 2026-05-11

**Authors:** Soyoung Shin, Wei Gao

**Affiliations:** ^1^ Andrew and Peggy Cherng Department of Medical Engineering Division of Engineering and Applied Science California Institute of Technology Pasadena California USA

**Keywords:** microfluidic architectures, sweat induction, sweat physiology, sweat transport

## Abstract

Sweat has emerged as a promising biofluid for wearable health monitoring due to its ability to provide noninvasive and continuous access to a wide range of biomarkers. While advances in sensing materials and device integration have accelerated the field, measurement reliability is fundamentally governed by how sweat is induced, intercepted, transported, and refreshed at the skin–device interface. Outside of exercise or thermal stress, sweat secretion is often low, transient, and spatially heterogeneous, making sweat availability—not sensing chemistry—the primary constraint for continuous monitoring. This Review reframes sweat sampling as an integrated engineering pipeline spanning physiological induction, on‐skin capture, directional transport, flow regulation, storage, and outlet‐driven refreshment. We summarize sweat gland physiology and compositional dynamics that define biomarker accessibility, compare whole‐body, localized thermal, and cholinergic induction strategies, and analyze modern microfluidic architectures designed to sustain temporal resolution under low and variable sweat flux. Particular emphasis is placed on system‐level coupling between programmable induction and controlled fluid handling to enable quantitative and long‐duration monitoring. By shifting focus from sensing chemistry alone to induction–sampling integration, we outline design principles required to make sweat a reliable and comparable biofluid for real‐world wearable applications.

## Graphical abstract

Wearable sweat sensing relies not only on chemical detection but also on controlled biofluid management. This Review integrates sweat physiology, induction strategies, and microfluidic sampling architectures, demonstrating how flux, transport, and refreshment shape measurement reliability. It presents sweat sampling as a coordinated engineering system enabling robust, quantitative, and long‐duration monitoring.

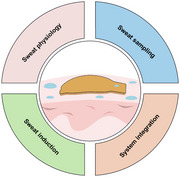

## 1 Introduction

Sweat is an attractive biofluid for wearable health monitoring because it enables noninvasive and continuous access to a broad range of molecular information. While blood remains the gold standard for systemic diagnostics and interstitial fluid (ISF) has gained traction through minimally invasive microneedle and continuous glucose monitoring platforms, both biofluids require skin penetration and are therefore constrained in wear duration, user compliance, and scalability. Many biomarkers traditionally assessed in blood or ISF—including electrolytes, metabolites, hormones, and small molecules—are also present in sweat, positioning it as a complementary biofluid for accessing systemic and local physiological information. The breadth of detectable biomarkers, combined with noninvasive and continuous sampling, enables wearable biochemical monitoring of hydration, metabolism, stress, and environmental exposures in ways that are difficult to achieve with blood‐ or ISF‐based approaches.

However, while continuous sweat sampling is possible in principle, the quality and temporal accuracy of biochemical measurements vary strongly with physiological and environmental conditions, particularly outside exercise or thermally stimulated states. Reliable sweat sensing depends not only on detecting analytes but also on controlling sweat induction, transport, and replacement within the device. Without such control, measurements may reflect accumulated or stagnant sweat rather than real‐time physiology. Although wearable sweat sensing has progressed substantially in materials, transduction, and system integration [1, 2, 3, 4, 5, 6, 7], measurement reliability ultimately depends on how sweat is accessed and handled. In many systems, sweat availability is assumed rather than engineered, and sampling is treated as a passive precursor to sensing. Yet because sweat secretion is intermittent, spatially heterogeneous, and strongly condition‐dependent, the induction and microfluidic management of sweat often dominate temporal accuracy and quantitative interpretation [7, 8].

Controlled sweat induction and quantitative sweat testing have long been established in clinical settings through sudomotor function assessments such as the quantitative sudomotor axon reflex test (QSART) and thermoregulatory sweat testing [9]. These protocols demonstrate that reproducible sweat stimulation and measurement are achievable when induction methods, timing, and readout strategies are explicitly designed around gland physiology. More recently, microfluidic‐focused reviews have drawn attention to sweat transport, filling dynamics, and flow‐rate measurement as important contributors to meaningful sweat analytics [10, 11].

As the field moves toward passive, long‐duration, and low‐sweat‐rate monitoring, a systematic examination of sweat induction and sampling as an integrated engineering pipeline is needed. Bridging physiological understanding with wearable device design is essential to clarify when sweat‐based measurements can be considered reliable and comparable across studies.

**FIGURE 1:**
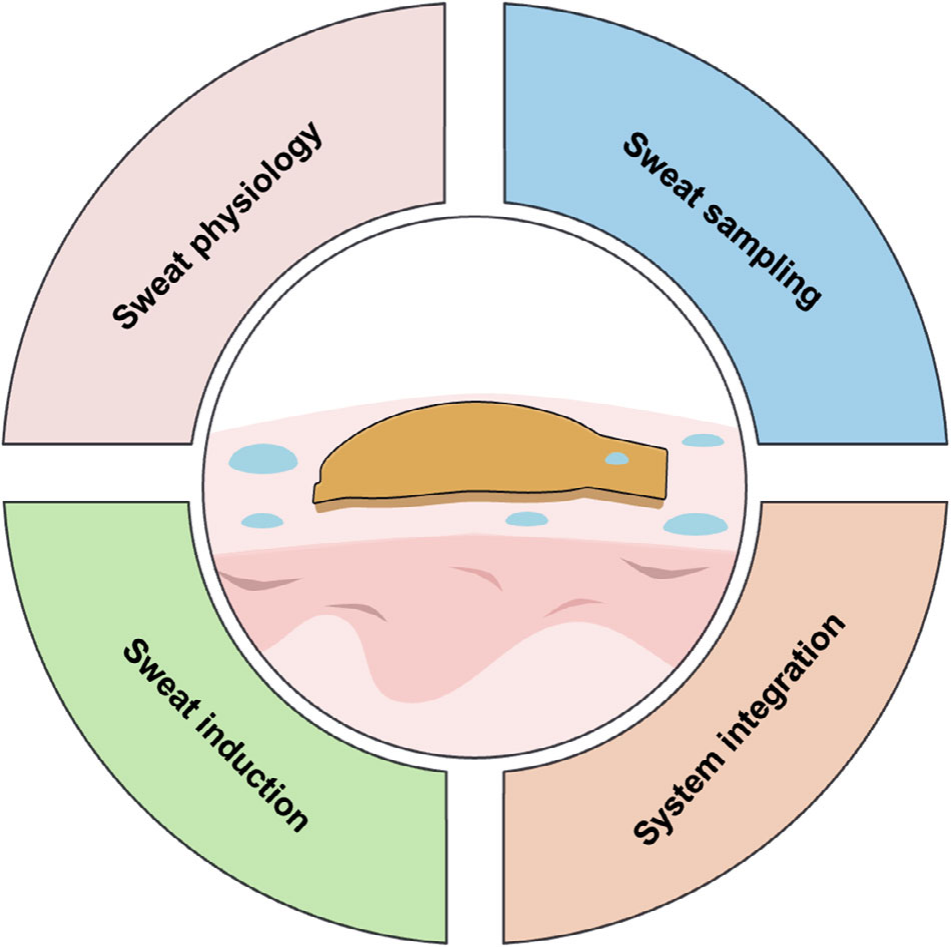
Integrated pipeline of sweat‐based wearable sensing. The framework links sweat physiology (gland distribution and composition), induction modalities (physiological and pharmacological activation), on‐skin sampling architectures (capture, transport, gating, and refreshment), and system integration. Reliable monitoring emerges from coordinated control across these interconnected layers.

In this Review, we frame sweat sampling as a pipeline spanning induction, harvesting at the skin–device interface, on‐skin transport, flow regulation, and refreshment (Figure 1). We first summarize sweat gland physiology to define how gland type, location, and sweat rate shape availability and composition (Section 2). We then compare physiological, thermal, and cholinergic induction strategies and their compositional implications (Section 3). Building on this foundation, we analyze modern on‐skin sampling architectures—including capture, directional transport, gating, storage, and outlet‐driven refreshment—with emphasis on sustaining temporal resolution under low and variable flux (Section 4). Finally, we discuss design principles and future directions for enabling reliable, passive, and long‐duration sweat monitoring (Section 5).

## 2 Sweat Physiology

### 2.1 Sweat Gland Types, Distribution, and Physiological Control

**FIGURE 2:**
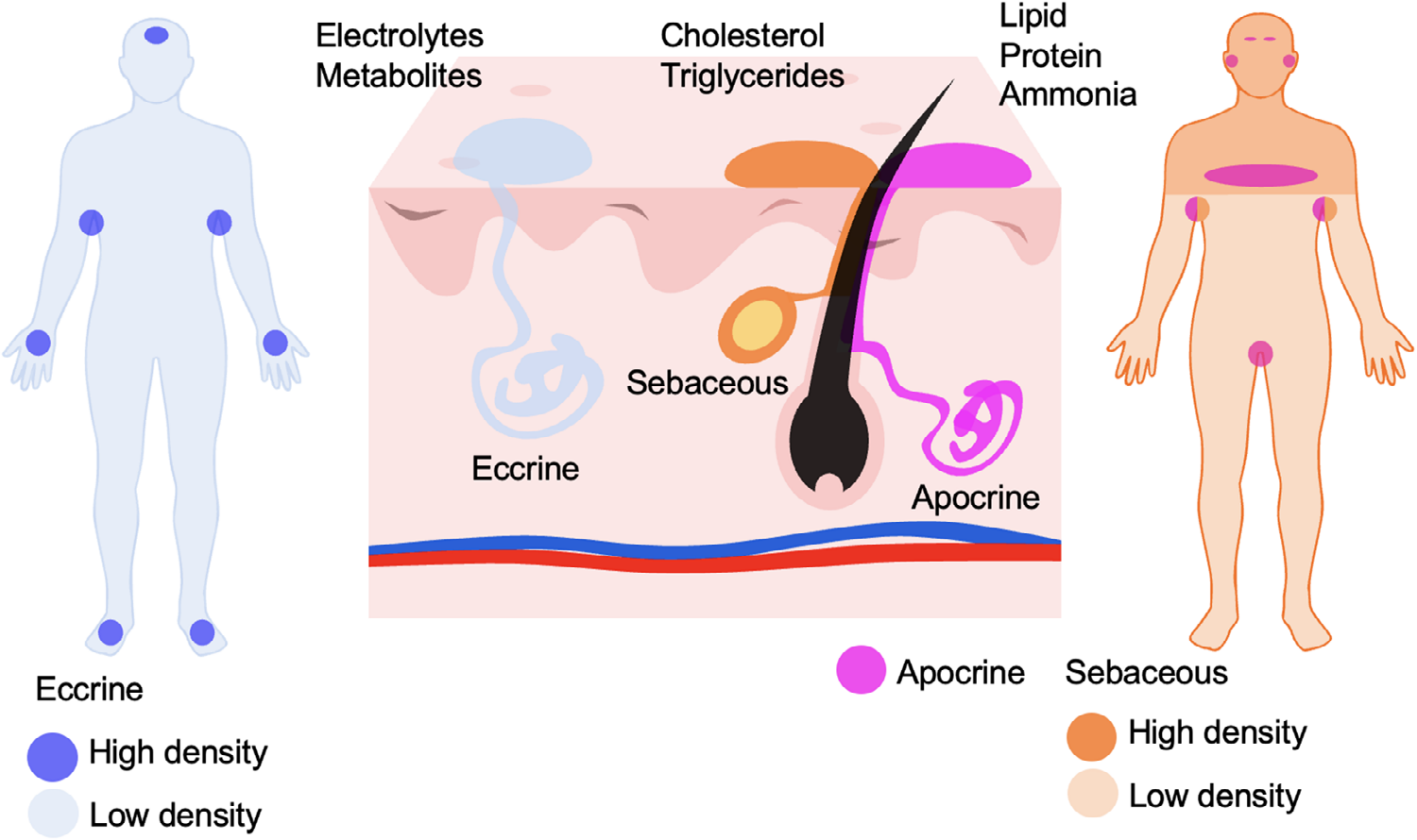
Glandular and regional determinants of sweat availability and composition. Eccrine, apocrine, and sebaceous glands differ in structure, control mechanisms, and secreted constituents. Their anatomical distribution and density variation across body regions generate spatially heterogeneous sweat flux and surface lipid environments, which directly influence wearable sweat interception, transport, and analyte partitioning.

Human skin contains multiple secretory appendages with distinct anatomical structures, spatial distributions, and regulatory mechanisms, collectively defining the boundary conditions for wearable sweat sampling (Figure 2). Among these, eccrine sweat glands are the dominant contributors to measurable sweat across most anatomical sites and therefore establish the primary physiological framework for wearable sensing.

Eccrine glands are widely distributed over the body surface, with an estimated ∼2–4 million glands in humans and strong site‐to‐site density variation (∼16–530 glands cm^−^^2^ depending on anatomical location) [12, 13, 14, 15]. Each gland consists of a coiled secretory unit connected to a reabsorptive duct, whose epithelial cell populations regulate fluid secretion and ion transport [16, 17]. Eccrine sweating is controlled predominantly by sympathetic cholinergic pathways and responds dynamically to thermal load, exercise, and pharmacological stimulation [18].

In contrast, apocrine sweat glands are localized to specific hair‐bearing regions—including the axilla, anogenital area, areola, and periumbilical skin—at much lower densities (typically ≤50 glands cm^−^^2^) [20]. These larger glands open into hair follicles and become active after puberty. Apocrine secretion occurs via a decapitation mechanism in which apical cytoplasmic portions of secretory cells are released into the follicular canal. Compared to eccrine sweat, apocrine secretions are richer in proteins, lipids, and steroidal compounds and are regulated primarily through adrenergic pathways, often in response to emotional or stress‐related stimuli [15, 21, 22]. A related subtype, the apoeccrine gland—described primarily in axillary skin—exhibits morphological and functional characteristics intermediate between eccrine and apocrine glands and can contribute substantially to local sweat output in this region [15, 23, 24]. These glandular differences become particularly relevant for location‐specific wearable studies, where regional secretion profiles may influence measured analyte composition.

Sebaceous glands, although not sweat glands, play an important role in shaping the chemical and physical environment at the skin surface. These lipid‐secreting appendages are associated with hair follicles and release sebum via a holocrine mechanism [25, 26]. Sebaceous gland density is especially high on the scalp and forehead (∼400–900 glands cm^−^^2^) and substantially lower on the limbs [25]. Sebum consists primarily of free fatty acids, wax esters, triglycerides, and squalene [27, 28], forming a lipid‐rich surface layer that mixes with aqueous sweat after secretion. While sebaceous glands do not generate sweat, their secretions modify surface wetting, evaporation dynamics, and analyte partitioning—factors that directly affect sweat interception and transport in wearable systems [25, 26]. Gland type, spatial distribution, and neural regulation together determine how sweat varies across the skin in both location and flow rate, which wearable sampling systems must accommodate.

### 2.2 Sweat Composition and Gland‐Specific Contributions

Beyond gland anatomy, the biochemical composition of sweat—and its dependence on secretion rate and glandular origin—directly constrains what can be quantitatively accessed through wearable sampling (Table 1). Although many analytes present in blood or interstitial fluid are also detectable in sweat, their concentrations and dynamics are governed by sweat‐specific physiological processes rather than simple plasma equilibration [29, 30, 31, 32].

**TABLE 1:** Sweat composition and gland‐specific contributions.

Biomarker	Class	Dominant gland / source	Sweat or skin‐surface concentration range	Blood concentration range	Sampling‐relevant notes
Sodium (Na^+^)	Electrolyte	Eccrine	∼10–90 mm	∼135–145 mm	Strong sweat‐rate dependence due to ductal reabsorption [14, 29, 34, 35, 157, 158]
Chloride (Cl^−^)	Electrolyte	Eccrine	∼10–80 mm	∼95–105 mm	Closely coupled to Na^+^ transport [157, 158, 159]
Potassium (K^+^)	Electrolyte	Eccrine	∼3–15 mm	∼3.5–5.0 mm	Less reabsorbed than Na^+^; weaker sweat‐rate dependence [157, 158, 159]
Lactate	Metabolite	Eccrine	∼5–50 mm	∼0.5–2 mm	Produced locally in gland; limited blood correlation [37, 159, 160]
Urea	Metabolite	Eccrine	∼2–20 mm	∼2.5–7.8 mm	Diffuses from blood; often enriched in sweat [38, 48]
Ammonia	Nitrogenous	Eccrine	∼0.1–5 mm	∼20–50 µm	pH‐dependent; partly from urea breakdown and local metabolism [37, 48]
Glucose	Metabolite	Eccrine	∼0.01–0.5 mm	∼4–6 mm	Low concentration; sensitive to sweat rate and contamination [159, 161]
Uric acid	Purine metabolite	Eccrine	∼5–60 µm	∼150–450 µm	Flow‐dependent; influenced by skin surface effects [30, 159]
Cortisol	Hormone	Mixed (eccrine + surface partitioning)	∼1–20 ng mL^−1^	∼50–200 ng mL^−1^	Region‐dependent; delayed relative to plasma [31, 102, 160, 162]
Testosterone	Steroid hormone	Apocrine‐associated	∼0.1–10 ng mL^−1^ (axillary sweat)	∼300–1000 ng dL^−1^	Site‐specific; enriched in apocrine regions [32, 163]
Androstenol / Androstenone	Steroid derivatives	Apocrine	nm–µm	Low / variable	Secreted as odor precursors; microbially transformed [42, 43, 164]
Free fatty acids	Lipids	Sebaceous (surface)	µg cm^−2^ (skin surface)	—	Mix with sweat post‐secretion; alter surface wettability [44, 45]
Squalene	Lipid	Sebaceous	∼5–15% of sebum lipids	—	Contributes to hydrophobic skin‐surface film; influences wetting and evaporation [27, 44]

*Note*: Values represent literature‐reported ranges or representative values under specific experimental conditions. Reported concentrations may vary depending on sweat rate, body location, and sampling methodology.

For eccrine‐derived electrolytes such as sodium and chloride, sweat concentration is strongly coupled to sweat rate through ductal reabsorption. At low secretion rates, prolonged residence time within the duct allows extensive ion reabsorption, resulting in lower sodium and chloride concentrations. As sweat rate increases, reabsorption efficiency decreases, and electrolyte levels rise accordingly [29, 33, 34, 35]. Potassium undergoes comparatively less reabsorption and exhibits weaker sweat‐rate dependence [29, 36]. These rate‐dependent shifts imply that measured electrolyte concentrations reflect not only systemic status but also local secretion dynamics—highlighting the need to interpret sensor output within the context of fluid flux.

Other eccrine‐associated analytes—including lactate, urea, ammonia, and uric acid—arise from a combination of local gland metabolism, diffusion from blood, and post‐secretion surface interactions [29, 30, 37, 38]. Lactate, for example, is produced within eccrine glands and can reach millimolar concentrations in sweat despite substantially lower plasma levels [37, 39, 40, 41]. Consequently, temporal changes in sweat metabolite levels may reflect glandular physiology and sweat rate modulation rather than direct systemic fluctuations.

Apocrine glands contribute a distinct molecular profile, particularly in axillary regions, including steroid hormones, proteinaceous components, and odor precursor compounds [23, 24, 42, 43]. These secretions are subject to microbial transformation at the skin surface, further modifying detectable chemical signatures. Region‐specific wearable measurements must therefore account for glandular heterogeneity and microbiome interactions.

Sebaceous secretions introduce an additional layer of compositional complexity [27, 44, 45]. Lipid‐rich sebum can mix with sweat at the surface, altering wetting behavior, evaporation rates, and analyte partitioning. Lipophilic hormones and xenobiotics may preferentially associate with this surface lipid reservoir rather than remain in the aqueous phase [28, 44]. As a result, measured concentrations of hydrophobic species may depend as much on surface chemistry as on glandular secretion.

## 3 Sweat Induction Methods

**FIGURE 3:**
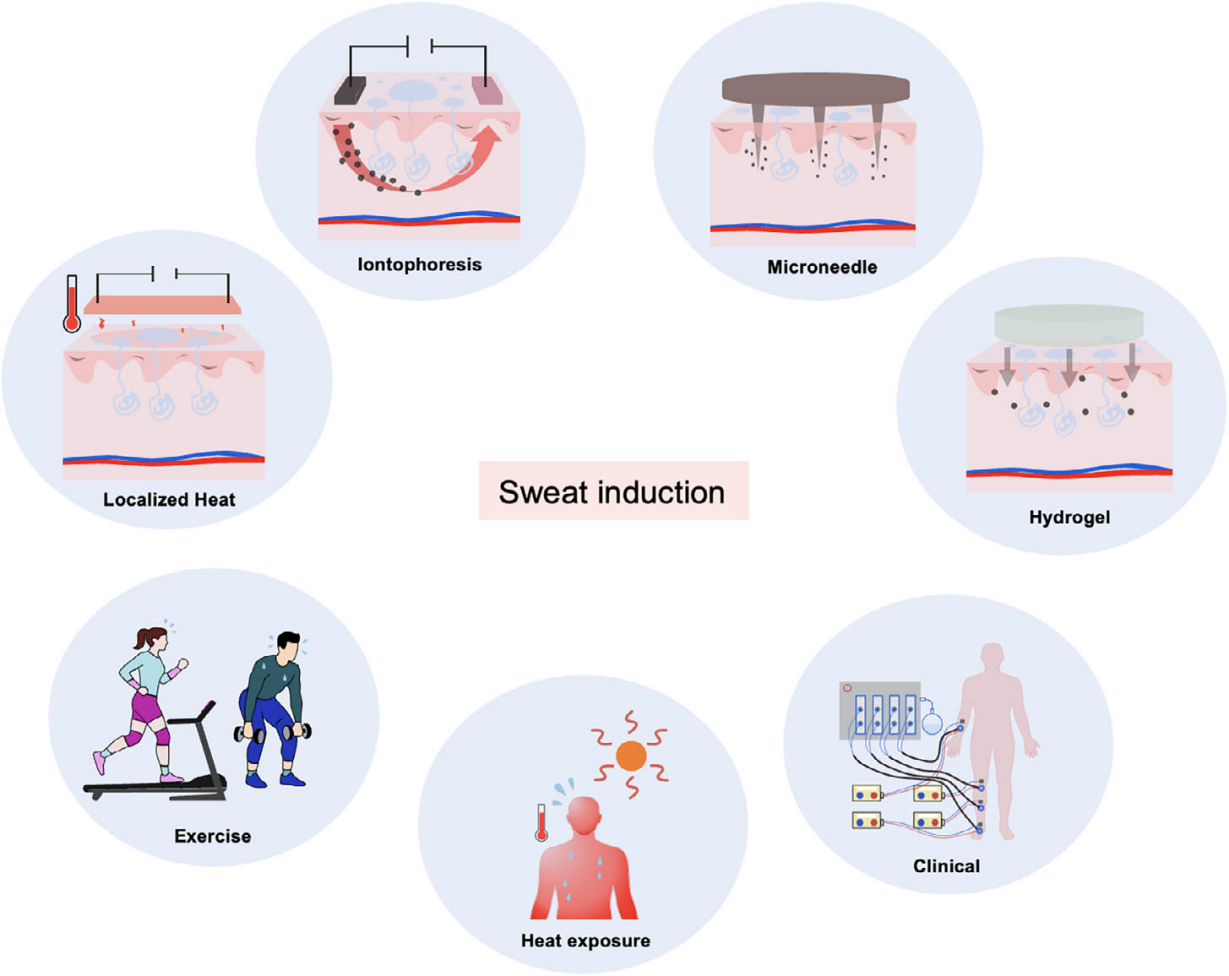
Overview of sweat induction strategies across physiological and device‐assisted modalities. Representative schematics of whole‐body induction (exercise and heat exposure), localized thermal stimulation, clinical testing protocols, and pharmacological activation via iontophoresis, microneedle‐assisted delivery, and hydrogel‐based interfaces. These modalities generate distinct sweat‐rate regimes, onset dynamics, and compositional conditions that directly influence downstream sampling architecture.

Sweat induction determines when, where, and at what rate fluid becomes available for sampling, thereby defining both the quantity and chemical characteristics of measurable sweat. Because sweat secretion is inherently condition‐dependent, the induction strategy directly shapes temporal resolution, compositional interpretation, and device‐level fluid management requirements. Existing approaches span a continuum from whole‐body physiological stimulation to highly localized, device‐assisted, and pharmacological strategies (Figure 3). These methods stimulate eccrine glands in different ways and lead to distinct sweat rates, response times, and compositions, which should be considered when comparing wearable systems.

### 3.1 Physiological and Localized Sweat Induction

#### 3.1.1 Whole‐Body Physiological Induction

Whole‐body induction—through exercise or passive heat exposure—stimulates sweating via central thermoregulatory pathways. Elevations in core and skin temperature activate sympathetic cholinergic innervation of eccrine glands, resulting in widespread secretion across large skin areas [40, 46]. Under peak conditions, sweat rates can exceed several mg·cm^−^^2^·min^−^^1^, though values vary substantially with fitness level, acclimatization, environmental conditions, and anatomical location [13, 47].

High sweat flux alters composition through ductal transport dynamics. Increased secretion shortens residence time within the reabsorptive duct, reducing electrolyte reabsorption and elevating sodium and chloride concentrations relative to low‐flow conditions [38, 40]. Exercise‐induced sweating may also shift sweat pH due to systemic acid–base changes and metabolic byproduct accumulation [48]. Thus, while whole‐body induction provides abundant fluid for sampling, it introduces physiologically coupled variability in sweat chemistry that complicates quantitative interpretation.

#### 3.1.2 Localized Thermal Induction

Localized heating offers spatially confined stimulation by elevating skin temperature at selected sites without significantly altering core temperature. This can be achieved using external heat sources or on‐skin electrothermal elements such as Joule heaters [49, 50, 51]. Localized thermal induction typically produces lower sweat rates than vigorous exercise but can generate sufficient flux for regional sampling while maintaining greater compatibility with wearable integration.

Because systemic thermoregulatory engagement is limited, localized heating partially decouples sweat availability from whole‐body physiological stress. However, electrolyte concentrations remain sweat‐rate dependent due to ductal reabsorption kinetics, and composition varies with stimulation intensity and duration [52]. Even under localized induction, therefore, secretion rate and fluid handling remain tightly linked.

### 3.2 Cholinergic Sweat Induction and Delivery Interfaces

Cholinergic induction strategies activate eccrine glands by mimicking endogenous acetylcholine signaling at muscarinic receptors (primarily M3) on secretory cells. These approaches enable localized and temporally controlled sweat generation without exercise or thermal stress and have become central to both clinical testing and wearable research.

#### 3.2.1 Cholinergic Agonists and Stimulation Characteristics

Pilocarpine is the most extensively used iontophoretic sweat inducer and remains the clinical standard for sweat testing, particularly in the diagnosis of cystic fibrosis. As a direct muscarinic receptor agonist with high affinity for M3 receptors, pilocarpine effectively stimulates eccrine sweat secretion when delivered into the viable epidermis and upper dermis [53, 54]. Compared with endogenous acetylcholine, pilocarpine is resistant to rapid enzymatic degradation, enabling sustained sweat production following a brief stimulation period.

Other cholinergic agents, including carbachol and acetylcholine analogs, have been explored to modulate stimulation strength, duration, and localization. Carbachol exhibits slower degradation and prolonged activity relative to pilocarpine, often producing longer‐lasting sweat responses at lower delivered doses [55]. Acetylcholine itself produces short‐lived stimulation due to rapid hydrolysis by acetylcholinesterase and is therefore less commonly used as a standalone inducer. The characteristics of commonly used iontophoretic agents, including their mechanisms of action, relative potency, and practical advantages and limitations, are summarized in Table 2.

**TABLE 2:** Comparison of Cholinergic Agents for Pharmacologic Sweat Induction.

Drug	Mechanism of Action	Relative Sweat Output	Effective Penetration / Depth	Key Advantages	Limitations
Pilocarpine	Direct cholinergic agonist activating eccrine secretory cells	Moderate–High; transient	Dermal eccrine gland level	Clinically validated; reproducible response; established safety profile	Transient stimulation (∼20–30 min under standard protocols) [53, 63]
Carbachol	Synthetic cholinergic agonist resistant to acetylcholinesterase degradation	High; sustained > pilocarpine	Dermal eccrine gland level	Longer‐lasting stimulation; effective at lower doses	Less clinically standardized; Potential receptor desensitization with prolonged stimulation [55]
Acetylcholine (ACh)	Endogenous cholinergic neurotransmitter; rapidly degraded by acetylcholinesterase.	Low–Moderate; rapid decay	Dermal eccrine gland level (limited diffusion radius)	Physiologically native; rapid degradation enables precise temporal control.	Rapid enzymatic degradation; short duration; rarely used alone [56]
ACh analogs / cholinergic esters	Synthetic cholinergic agonists engineered for enhanced stability and modified degradation kinetics.	Variable; tunable	Dermal eccrine gland level	Adjustable degradation kinetics; potential for tailored stimulation profiles	Limited clinical validation; regulatory uncertainty [55, 165]

*Note*: Data are compiled from representative literature sources under specific experimental or clinical conditions. Reported performance may vary depending on dosage, delivery method, and measurement protocol.

#### 3.2.2 Iontophoretic Delivery

Iontophoresis is the most established method for delivering cholinergic agonists transdermally. By applying a low‐level electric current, charged molecules are transported across the stratum corneum to activate eccrine glands locally [53, 56]. This electrically assisted delivery provides site‐specific, controllable stimulation and avoids the need for systemic thermal activation.

Iontophoretic induction typically produces moderate, localized sweat rates that are lower than those observed during intense exercise but higher and more controllable than passive resting conditions [57, 58]. Because sweat rates under iontophoresis are often sustained but not extreme, electrolyte concentrations measured under these conditions frequently reflect intermediate levels of ductal reabsorption compared with exercise‐induced sweating [38]. Local sweat pH may also be influenced by the delivered drug, applied current density, and stimulation duration, highlighting the importance of considering induction conditions when interpreting pH‐sensitive measurements.

#### 3.2.3 Alternative Delivery Interfaces

In contrast to conventional iontophoresis, emerging strategies seek localized sweat induction through alternative delivery interfaces or powering mechanisms that minimize sustained direct‐current transport. Microneedle‐assisted platforms mechanically bypass the stratum corneum to introduce cholinergic agonists directly into the superficial dermis [59, 60, 61]. Solid microneedles create transient microscale conduits that enable diffusion‐dominated drug transport from an integrated reservoir or coating [59], whereas hollow microneedles permit direct microinjection of pilocarpine solutions into superficial tissue layers [60, 61]. Replacing electromigration with mechanically assisted delivery maintains spatially confined gland stimulation and may reduce irritation associated with prolonged electrical exposure.

Self‐powered systems provide another route toward autonomous induction. Triboelectric nanogenerators (TENGs) convert biomechanical motion into transient electrical outputs capable of driving mild iontophoretic or electro‐stimulatory effects [62]. Although the mechanism remains electrically mediated, such systems remove dependence on external power sources and support fully integrated wearable platforms.

### 3.3 Induction‐Dependent Effects on Sweat Rate and Composition

Induction modality directly determines both sweat flux and the chemical environment at the skin surface. Whole‐body physiological stimulation generally produces the highest sweat rates, while localized thermal and pharmacological approaches generate lower, spatially confined secretion [40]. Differences in flux translate into systematic shifts in electrolyte concentration due to sweat‐rate–dependent ductal reabsorption, particularly for sodium and chloride [38, 40].

Sweat pH has also been shown to vary with induction method and sampling location. Exercise‐induced sweat is often reported to exhibit lower pH values than pharmacologically induced sweat, reflecting contributions from metabolic activity, systemic acid–base balance, and regional differences in gland activity [48]. Direct experimental comparisons have demonstrated statistically significant differences in sweat pH between exercise‐driven and iontophoretically induced sweat, underscoring that pH should be interpreted as a condition‐dependent parameter rather than an intrinsic property of sweat [58]. These findings show that measured sweat composition is influenced not only by the fluid itself but also by the conditions under which it is produced.

For wearable systems, the choice of induction method therefore sets the starting conditions for sampling. Sweat rate, response time, and rate‐dependent composition affect how the device should be designed, including inlet structure, channel resistance, refreshment, and how the data are interpreted.

To facilitate comparison across approaches, a summary of major sweat induction methods and their implications for wearable sampling is provided in Table 3.

**TABLE 3:** Comparison of sweat induction methods and implications for wearable sampling.

Induction method	Sweat‐rate regime	Spatial localization	Temporal control	Composition dependence	Suitability for long‐duration wear
Exercise / whole‐body heating	High; can exceed several mg cm^−^^2^ min^−^^1^	Whole body	Low (physiology‐driven)	Strong sweat‐rate dependence due to ductal reabsorption; pH and metabolites influenced by systemic physiology	Limited; requires sustained activity or thermal stress [13, 40]
Localized thermal stimulation	Moderate; lower than exercise but sufficient for regional sampling	Localized	Moderate (controlled heating)	Composition still sweat‐rate dependent; partially decoupled from systemic physiology	Moderate; compatible with wearable integration [49, 52]
Cholinergic iontophoresis (e.g., pilocarpine, carbachol)	Low–moderate; sustained but localized	Highly localized	High (programmable stimulation)	Drug‐dependent effects; intermediate electrolyte levels; possible pH shifts due to stimulation conditions	High; widely used for wearable and clinical applications [1, 55, 70]

*Note*: Characteristics are summarized from representative studies and may vary depending on experimental conditions and device design.

## 4 Modern On‐Skin Sampling Architectures: From Sweat Capture to Evaporation Control

Historically, sweat analysis relied on clinical and laboratory‐based batch collection methods, including pilocarpine‐based macroduct systems for cystic fibrosis diagnosis, absorbent pads and filter papers for exercise physiology studies, and ventilated capsule techniques for continuous local sweat rate measurement under controlled thermal conditions [53, 63, 64, 65]. While these approaches established foundational physiological insights, they are poorly suited for continuous wearable monitoring due to bulk instrumentation, limited temporal resolution, and lack of integrated fluid management.

**FIGURE 4:**
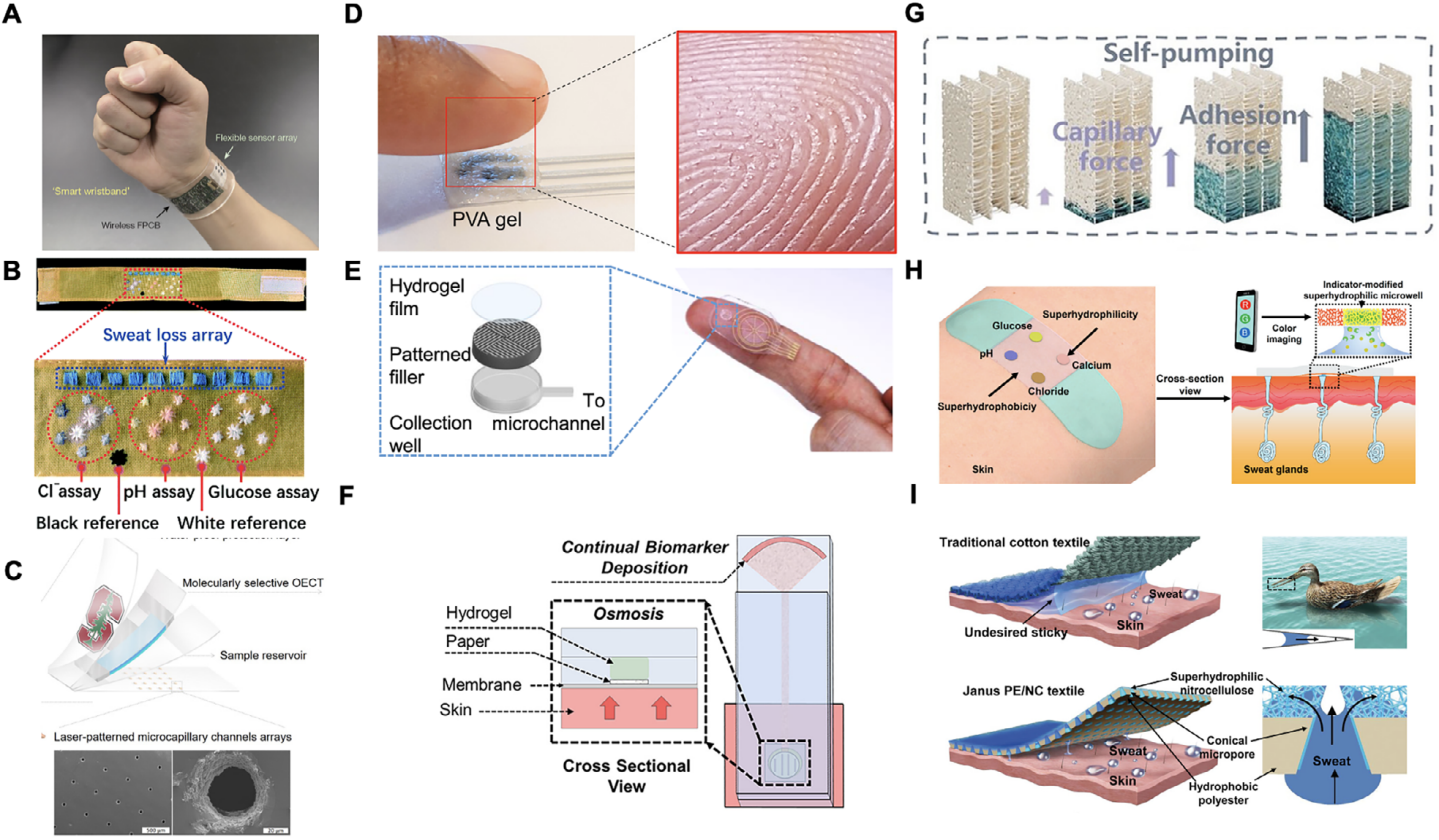
Strategies for initial sweat sampling at the skin–device interface. (A) Passive absorbent materials that collect sweat directly beneath sensing regions, enabling simple uptake but limited refreshment control. Reproduced with permission [68]. Copyright 2016, Springer Nature. (B) Thread‐ and fabric‐based wearable bands that provide conformal skin contact and distributed capillary wicking for sweat transport. Reproduced with permission [73]. Copyright 2021, Royal Society of Chemistry. (C) Microstructured capillary inlet arrays that expand the effective wetting perimeter, increasing interception efficiency under reduced secretion rates. Reproduced with permission [77]. Copyright 2018, American Association for the Advancement of Science. (D–F) Hydrogel‐based interfaces that enhance sweat uptake through interconnected micro‐ and nanoscale polymer networks, supporting improved absorption and buffering capacity. (D) Reproduced with permission [88]. Copyright 2021, American Chemical Society. (E) Reproduced under the terms of the CC‐BY license [83]. Copyright 2021, Nyein et al., published by Springer Nature. (F) Reproduced with permission [84]. Copyright 2021, American Chemical Society. (G) Hierarchically porous biosponge collectors with increased void volume and permeability to reduce hydraulic resistance and accelerate fluid entry. Reproduced under the terms of the CC‐BY license [89]. Copyright 2024, Ding et al., published by Wiley‐VCH GmbH. (H) Planar Janus interfaces employing in‐plane wettability gradients to bias directional transport at the skin boundary. Reproduced with permission [99]. Copyright 2019, American Chemical Society. (I) Three‐dimensional Janus architectures that combine wettability gradients with geometric asymmetry to enforce unidirectional skin‐to‐inlet flow and improve refreshment under low and variable sweat rates. Reproduced with permission [90]. Copyright 2017, Wiley‐VCH GmbH.

**FIGURE 5:**
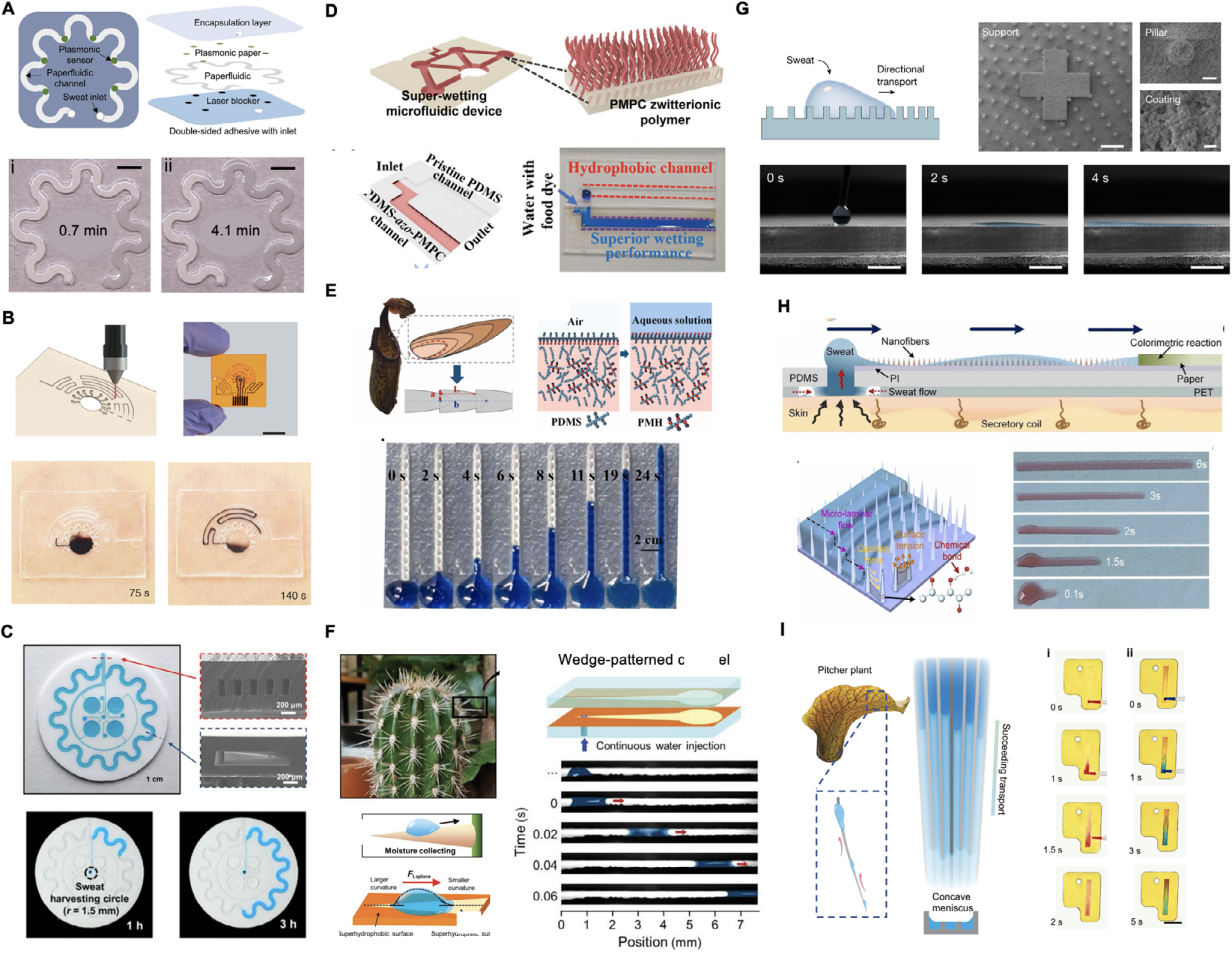
Self‐driven microfluidic strategies for directional sweat transport from the inlet to sensing regions. (A) Paper‐based microfluidics utilizing distributed absorbent networks for capillary‐driven sweat transport. Reproduced under the terms of the CC‐BY license [104]. Copyright 2022, Mogera et al., published by AAAS. (B, C) Defined microfluidic channels fabricated by laser‐based methods or soft elastomeric molding to guide sweat through enclosed or open pathways. (B) Reproduced with permission [114]. Copyright, 2020 Springer Nature. (C) Reproduced with permission [67]. Copyright 2016, American Association for the Advancement of Science. (D) Surface‐modified microfluidic channels incorporating hydrophilic coatings. Reproduced with permission [117]. Copyright 2022, Wiley‐VCH Verlag GmbH & Co. KGaA. (E, F) Bioinspired microfluidic architectures featuring wettability gradients and structural asymmetry, including Nepenthes‐, duck‐tongue–, cnidarian tentacle–, and cactus spine–inspired designs. (E) Reproduced with permission [120]. Copyright 2026, Elsevier B.V. (F) Reproduced with permission [124]. Copyright 2021, Wiley‐VCH Verlag GmbH & Co. KGaA. (G–I) Three‐dimensional microfluidic architectures for directional sweat transport. (G) Liquid‐diode systems, Reproduced with permission [129]. Copyright 2024, Springer Nature. (H) nanofiber‐based transport networks, Reproduced with permission [130]. Copyright 2024, Elsevier. and (I) hierarchical and gradient‐guided microchannel architectures. Reproduced under the terms of the CC‐BY license [97]. Copyright 2025, Shin et al., published by the American Association for the Advancement of Science.

**FIGURE 6:**
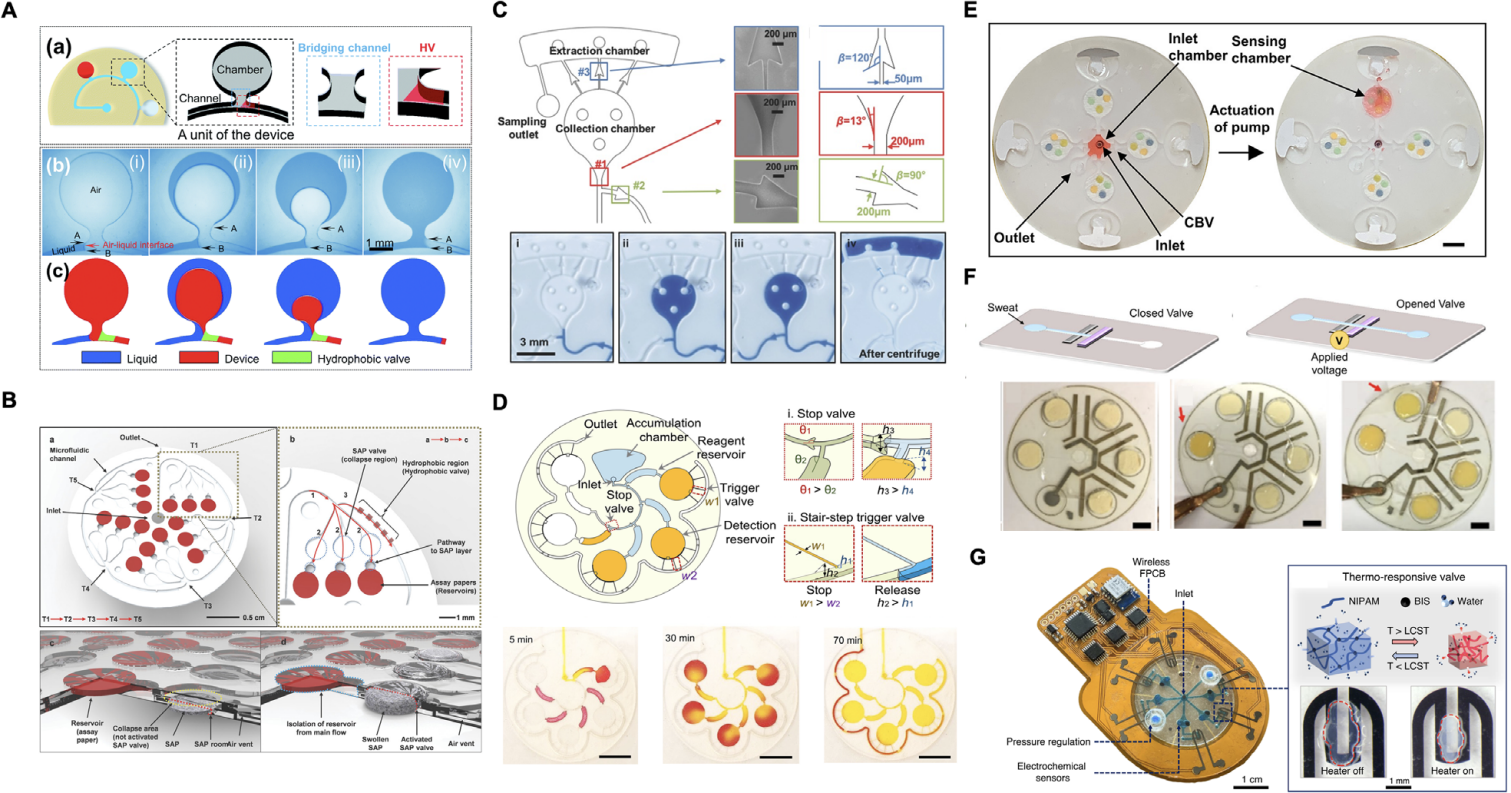
Flow gating, sequencing, and storage architectures for time‐resolved sweat sampling. (A) Hydrophobic stop valves based on localized surface treatment for reservoir isolation. Reproduced with permission [131]. Copyright 2020, Royal Society of Chemistry. (B) Hydrophobic stop valves coupled with superabsorbent polymer (SAP) collapse elements. Reproduced with permission [132]. Copyright 2018, Wiley‐VCH Verlag GmbH & Co. KGaA. (C) Capillary bursting valves defined by geometry‐modulated pressure thresholds. Reproduced with permission [136]. Copyright 2017, Wiley‐VCH Verlag GmbH & Co. KGaA. (D) Three‐dimensional bursting‐valve architectures incorporating height variation or multilayer structures. Reproduced under the terms of the CC‐BY license [139]. Copyright 2025, Tu et al., published by the American Association for the Advancement of Science. (E–G) Programmable valving systems for externally regulated sweat routing, including (E) mechanically actuated valves, Reproduced with permission [142]. Copyright 2022, American Chemical Society. (F) electrowetting‐based microvalves, Reproduced with permission [143]. Copyright 2021, Springer‐Verlag GmbH Germany, part of Springer Nature, and (G) thermoresponsive hydrogel (PNIPAM) valve. Reproduced under the terms of the CC‐BY license [144]. Copyright 2020, Lin et al., published by Springer Nature.

To address these limitations, modern wearable sweat systems have evolved toward integrated on‐skin sampling architectures that actively regulate sweat interception, transport, storage, and refreshment under low and variable secretion conditions. Rather than treating fluid access as incidental, these platforms encode fluid management directly into device structure. This section examines these strategies sequentially—from inlet to outlet—while preserving representative design examples (Figures 4, 5, 6).

### 4.1 Sweat Capture and Entry Control

The inlet governs whether secreted sweat is effectively intercepted or lost to evaporation and lateral spreading at the skin–device interface. It therefore defines the first boundary condition of wearable sampling. Skin compatibility at the device interface is also critical for reliable sweat capture, as conformal contact and material properties can influence both sweat collection efficiency and user comfort during prolonged wear [66, 67, 68].

Early wearable sweat sensors commonly relied on passive absorbent materials placed directly in contact with the skin, such as pads or porous substrates, which enabled simple sweat uptake but offered limited control over saturation, refreshment, and spatial confinement of collected sweat [68, 69, 70, 71, 72]. For example, a fully integrated epidermal sensor array directly interfaced electrochemical electrodes with the skin surface, allowing sweat to accumulate beneath the sensing region without defined inlet confinement or active refreshment control (Figure 4A) [68]. Similarly, thread‐ and fabric‐based bands were introduced to wick sweat away from the skin through distributed capillary networks [73, 74, 75]. In one representative design, a textile‐based band integrated capillary thread channels with printed electrochemical sensors, relying on passive wicking along the fiber network to transport sweat from the skin to detection sites (Figure 4B) [73]. Although absorbent pads and textile‐based bands increase skin contact area and user comfort by passively wicking sweat away from the skin, their transport remains largely governed by symmetric capillary uptake, leading to bidirectional spreading, local pooling, and limited control over sweat residence time. As a result, while effective under high sweat rates, these wicking‐based configurations can experience delayed replacement of sampled sweat under low or intermittent secretion conditions.

Later designs expanded the effective wetting perimeter of the inlet through capillary‐enhanced microstructures, such as arrays of discrete microcapillaries or patterned surface features, to promote more efficient sweat entry from the skin [76, 77]. For instance, a molecularly selective nanoporous membrane‐based wearable organic electrochemical device employed a porous membrane layer to selectively admit sweat analytes while passively guiding sweat into the sensing region (Figure 4C) [77]. Increasing the number of contact points at the interface improves sweat capture at low secretion rates, though downstream flow remains symmetric without directional features.

Hydrogel‐based interfaces further enhanced sweat uptake by leveraging intrinsic micro‐ and nanoscale polymer networks [78, 79, 80, 81, 82, 83, 84, 85, 86, 87]. A touch‐based fingertip glucose monitoring platform used a porous hydrogel layer to rapidly absorb small volumes of eccrine sweat during brief contact (Figure 4D) [88]. Another example of a capillary‐enhanced hydrogel interface was integrated for sweat sampling to improve sweat interception and maintain reliable contact under low baseline secretion conditions (Figure 4E) [83]. Building on capillary‐driven uptake, osmotic–capillary patches incorporated hydrogel reservoirs designed to sustain continuous sweat extraction over extended wear durations (Figure 4F) [84]. Building on hydrogel‐enabled uptake, hierarchical porous matrices further reduce the hydraulic barrier to sweat interception. A nature‐inspired superhydrophilic biosponge integrates interconnected micro‐ and nanoscale fibrillar structures within the inlet, substantially increasing permeability and lowering the hydraulic pressure required for sweat entry (Figure 4G) [89]. Capillary‐enhanced microstructures improve vertical uptake and reduce collection time under low secretion conditions.

To provide directional transport at the skin–device boundary, asymmetric wettability was introduced. By creating surface‐energy gradients, these interfaces steer sweat toward the inlet while suppressing lateral spreading across the skin [90, 91, 92, 93, 94, 95, 96, 97, 98, 99]. Janus textile bands, for example, use a hydrophilic skin‐facing layer coupled to a more hydrophobic outer layer to enable spontaneous self‐pumping across the textile thickness (Figure 4H) [99]. In the integrated smart Janus textile band, this wettability contrast supports continuous and directional sweat sampling without external actuation, allowing sustained collection under low secretion conditions. To further strengthen directional control, bioinspired Janus textiles incorporate geometric asymmetry in addition to wettability contrast. Conical micropore architectures introduce curvature‐driven pressure differences that work with the surface‐energy gradient to promote unidirectional transport and suppress reverse flow (Figure 4I) [90]. Wettability gradients integrated within asymmetric geometries guide sweat from the skin into the inlet, supporting continuous refreshment under variable secretion rates.

### 4.2 Directional Transport and Flow Control

Once sweat enters the inlet, controlled transport through the device becomes essential to sustain continuous sampling. Channel design governs flow stability, residence time, and susceptibility to backflow. From a fluid mechanics perspective, sweat transport in wearable microfluidic systems is governed by capillary pressure, hydraulic resistance, and evaporation‐driven flux. These factors determine fluid velocity, residence time, and the ability to sustain continuous refreshment under low secretion conditions [100, 101, 102].

Early approaches relied on material‐driven capillary transport, in which porous substrates or paper‐based microfluidics wicked sweat toward sensing zones through distributed absorbent networks [103, 104, 105]. For example, colorimetric dermal patches used patterned absorbent networks to distribute sweat to discrete chambers without defined hydraulic resistance or rectification (Figure 5A) [104]. While simple and robust, such architectures are prone to uncontrolled lateral spreading and variable residence times under low sweat‐rate conditions.

To improve precision, defined microfluidic conduits were introduced. Enclosed channels fabricated through molding, soft lithography, or laser patterning allowed independent tuning of width, depth, and length, thereby modulating capillary pressure and flow resistance [67, 106, 107, 108, 109, 110, 111, 112, 113]. For instance, laser‐patterned wearable sensors guided sweat along predefined conduits toward sensing reservoirs (Figure 5B) [114], while soft lithography–based elastomeric devices created isolated microchannels that reduced cross‐contamination and improved volumetric control (Figure 5C) [67].

Despite these advantages, fluid transport within symmetric channels remained fundamentally isotropic, lacking intrinsic mechanisms for flow rectification. Although microfluidic systems do not purify sweat in the conventional sense, they can improve measurement accuracy by limiting contamination from the skin surface, controlling residence time within the device, and reducing mixing between newly secreted and previously collected sweat [83, 115, 116].

Surface chemistry modification further improved transport reliability [117, 118, 119, 120]. Hydrophilic and zwitterionic coatings decrease contact angle hysteresis and mitigate flow interruptions caused by protein adsorption or localized dehydration (Figure 5D) [117]. By stabilizing the liquid–solid interface, these treatments improve filling reliability under low and fluctuating sweat rates. Building upon surface‐energy optimization, two‐dimensional biomimetic microfluidic architectures began integrating geometric guidance with controlled surface treatment to further regulate sweat transport [120, 121, 122]. Inspired by natural wetting interfaces such as *Nepenthes alata*, these designs employ duck‐tongue‐like planar structures that promote preferential fluid spreading along defined pathways (Figure 5E) [120]. Geometric asymmetry combined with appropriate wettability guides fluid toward the sensing region while maintaining stable filling. To strengthen and extend directional transport, these designs incorporate wettability gradients along asymmetric channel geometries [123, 124, 125]. Cactus spine–inspired architectures exploit curvature‐induced Laplace pressure gradients and surface‐energy variation to promote spontaneous directional transport from inlet to sensing zones (Figure 5F) [124]. With geometry and wettability asymmetry, these systems move beyond passive capillary flow to enable built‐in directional transport.

More recently, three‐dimensional architectures incorporate structural features that promote directional flow [97, 126, 127, 128, 129, 130]. Liquid‐diode systems employ asymmetric pore geometries across the membrane thickness, establishing direction‐dependent capillary pressure barriers that permit forward transport while suppressing reverse flow (Figure 5G) [129]. Nanofiber‐based microchannels provide high‐surface‐area, low‐resistance pathways that promote rapid capillary uptake and redistribution under low sweat rates, enabling self‐driven and real‐time analysis without external pumping (Figure 5H) [130]. The interconnected fibrous network enhances capillary spreading and accelerates analyte delivery toward sensing regions without external pumping. In parallel, hierarchically guided microchannel architectures coordinate sweat interception, transport, and redistribution across multiple length scales to enable multiday operation (Figure 5I) [97]. Three‐dimensional fluid pathways maintain directional transport while minimizing stagnation and backflow under variable or fluctuating sweat rates. These strategies transition from planar guidance toward architecturally encoded fluid management, enabling sustained, robust sweat transport for continuous wearable monitoring.

### 4.3 Flow Gating, Sequencing, and Storage

In addition to directional transport, wearable sweat systems often incorporate flow‐gating mechanisms to regulate when and how fluid enters sensing reservoirs. Unlike continuous flow‐through designs, time‐resolved analysis benefits from controlled entry, temporary retention, and, in some cases, isolation of discrete sweat volumes. Flow‐gating strategies therefore directly influence sampling resolution, refresh dynamics, and resistance to back‐mixing under variable secretion conditions.

A widely adopted passive approach employs hydrophobic stop valves. Localized wettability contrast forms a capillary barrier that delays reservoir filling until upstream pressure exceeds the valve's opening pressure [131, 132, 133]. For example, patterned hydrophobic stop valves positioned at the reservoir inlets regulate sweat entry into discrete chambers (Figure 6A) [131]. The one‐opening chamber design spatially isolates collected sweat while preventing premature downstream flow. To improve robustness without external actuation, surface‐defined barriers have been combined with volume‐responsive materials such as superabsorbent polymers (SAPs), introducing mechanically assisted gating (Figure 6B) [132]. Upon swelling, SAP elements temporarily restrict downstream flow until a defined expansion volume is reached. These hybrid valves, which combine capillary‐driven flow with volume‐dependent mechanical response, enhance functional stability under intermittent secretion while remaining fully passive.

Burst pressure defined by channel geometry provides more predictable gating than surface‐ or material‐dependent mechanisms. Capillary bursting valves exploit abrupt channel expansions, cross‐sectional transitions, and expansion angles to establish defined bursting pressures governed by geometric control of the Laplace pressure barrier [134, 135, 136, 137, 138, 139, 140]. The sudden change in curvature at the expansion interface pins the liquid meniscus until the applied pressure exceeds the geometry‐dependent threshold. In planar microfluidic layouts, such geometric expansions are commonly implemented through lateral width transitions to enable sequential reservoir filling, allowing time‐resolved sampling (Figure 6C) [136]. Building on this principle, three‐dimensional structural modulation further expands the design space [138, 139, 141]. Incorporating height variation, multilayer stacking, or stepped channel profiles introduces vertical curvature transitions, allowing gating and release behavior to be programmed with greater precision and flexibility (Figure 6D) [139].

While passive gating relies on pre‐defined geometric or surface‐energy barriers to regulate flow autonomously, active strategies integrate actuation mechanisms that allow dynamic control over sweat routing and sampling timing [142, 143, 144, 145, 146]. Mechanical actuation represents one implementation of this approach. A soft wearable microfluidic patch with finger‐actuated pumps and valves allows users to initiate flow and selectively route sweat to designated reservoirs on demand (Figure 6E) [142]. Direct control of internal pressure and valve states enables both longitudinal and event‐triggered sampling within a single device. Electrical modulation provides another pathway for active control. Electrowetting‐based valves dynamically tune surface wettability through applied voltage, reversibly altering capillary thresholds to redirect sweat between channels (Figure 6F) [143]. This enables rapid and repeatable reconfiguration of flow paths without mechanical movement. More integrated epidermal systems further extend active gating through stimulus‐responsive materials embedded within soft microfluidic architectures (Figure 6G) [144]. In a programmable epidermal microfluidic valving system, thermoresponsive hydrogels integrated within PDMS microchannels modulate channel permeability through reversible swelling and deswelling. Temperature‐dependent volumetric changes alter local flow resistance, enabling programmable control of sweat routing and contextual biomarker analysis without mechanical components.

### 4.4 Outlet Engineering and Passive Pumping

While inlet design, channel transport, and flow gating have received substantial attention in wearable sweat microfluidics, limited examples have focused on outlet engineering and sink‐side control. Yet the outlet defines the downstream boundary condition of the fluidic system. It plays a critical role in sustaining forward flow, maintaining sweat refreshment, and minimizing backflow or stagnation—particularly under low and intermittent secretion. Without an effective sink, sweat accumulation within channels or reservoirs increases residence time, reduces temporal resolution, and elevates the risk of sample carryover.

Although this area remains comparatively less studied, evaporation‐assisted pumping is one of the most adopted strategies. Exposed liquid–air interfaces, porous substrates, or vapor‐permeable outlet regions enable controlled evaporation, which generates capillary pressure gradients that support continuous flow from upstream regions [147, 148, 149]. In this configuration, evaporation is not merely a passive loss mechanism but an active driving force that supports sustained transport and refreshment during extended wear. The resulting pressure differential promotes directional flow without additional mechanical components, making evaporation‐based designs particularly attractive for low‐power or fully passive systems.

Asymmetric membrane‐based outlets extend principles of directional wettability previously used in Janus transport architectures [97]. Porous membranes patterned with superhydrophilic and superhydrophobic regions allow sweat to exit while limiting reverse flow under motion, compression, or external pressure. When positioned at the device boundary, such membranes function as passive flow stabilizers, reinforcing unidirectional transport without introducing additional gating complexity.

In some systems, outlet interfaces are further engineered to enable periodic removal or resetting of accumulated sweat, preventing long‐term stagnation and preserving measurement fidelity during extended wear [145]. Despite recent advances, outlet engineering remains comparatively less developed compared to inlet and channel design, with limited examples reported for controlled fluid removal and refreshment in wearable systems [115, 150]. As wearable platforms advance toward longer‐duration and lower‐flux monitoring scenarios, deliberate sink‐side control will become increasingly important for sustaining continuous refreshment, limiting carryover, and ensuring quantitative reliability.

### 4.5 Coupling Induction and Sampling: System‐Level Design Considerations

Effective wearable sweat monitoring requires synchronization between the chosen induction modality and the downstream fluid‐handling capacity of the device. Induction defines when and how much sweat becomes available, while sampling architecture determines whether that fluid can be stably transported, refreshed, and measured. Mismatch between these two components often limits temporal precision and quantitative reliability.

**FIGURE 7:**
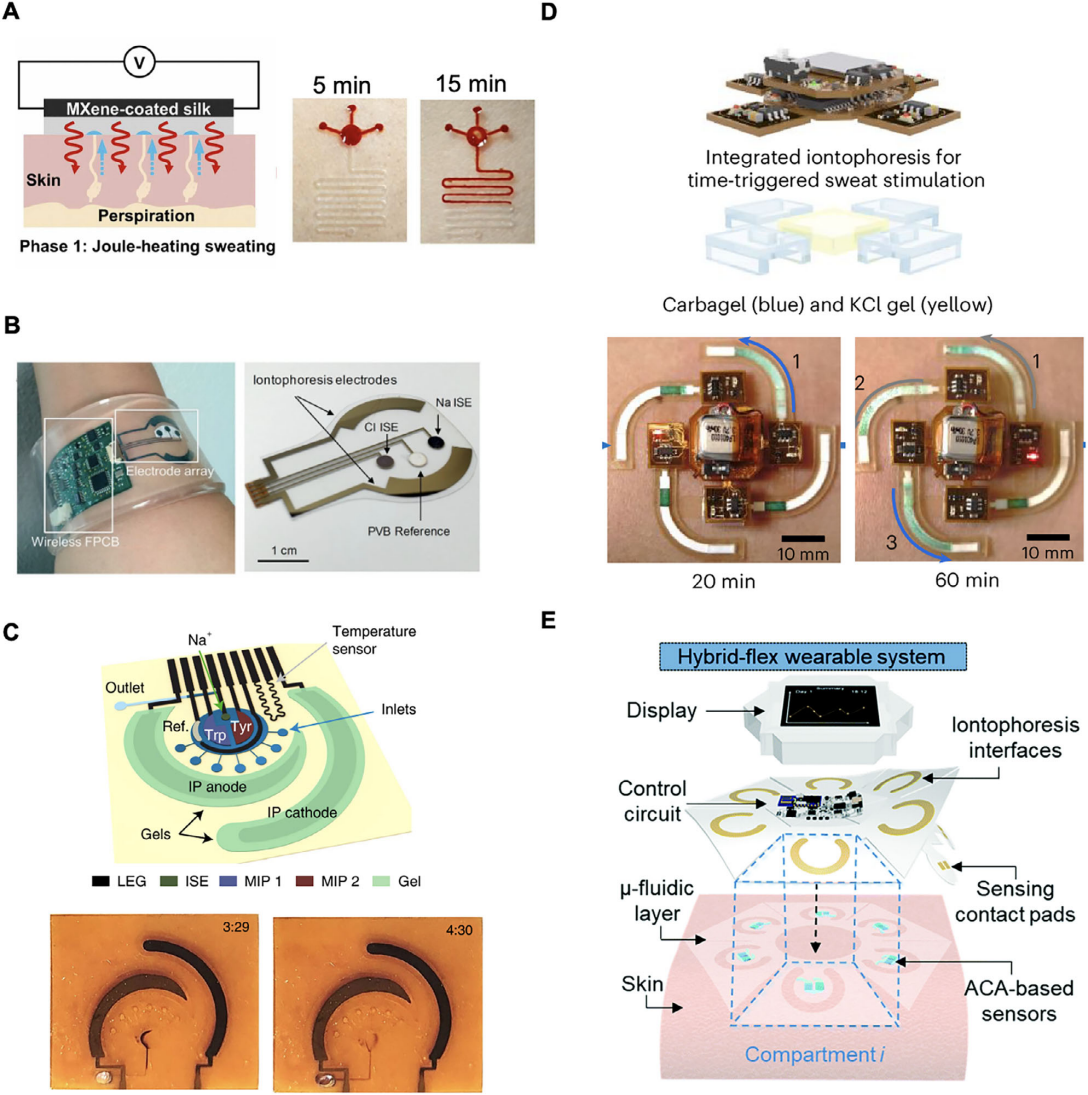
System‐level integration of sweat induction and sampling architectures. (A) Wearable patch employing localized Joule heating for sweat induction. Reproduced with permission [50]. Copyright 2024, Elsevier. (B) Integrated wearable system combining iontophoresis with on‐patch sweat analysis. Reproduced with permission [70]. Copyright 2017, PNAS. (C) Wearable platform integrating iontophoresis with microfluidic sampling and electrochemical sensing. Reproduced with permission [154]. Copyright 2022, Springer Nature. (D) Time‐sequenced multi‐site iontophoresis platform with electrochromic timing elements for time‐dynamic sweat sampling. Reproduced with permission [155]. Copyright 2026, Springer Nature. (E) Autonomous multi‐compartment wearable system with programmable iontophoresis for diurnal biomarker acquisition. Reproduced with permission [156]. Copyright 2020, Royal Society of Chemistry.

One strategy employs electrothermal stimulation to induce localized sweating without pharmacological agents. Wearable sensor patches integrating Joule heating–assisted sweating introduced electrothermal stimulation as an alternative induction modality (Figure 7A) [50]. Localized skin heating reduces reliance on cholinergic agents while directing induced sweat into adjacent microfluidic sampling regions. Because heating can be spatially confined and electronically controlled, electrothermal induction provides a straightforward pathway toward integrated stimulation–sampling architectures. This localized and moderate sweat flux imposes distinct constraints on microfluidic design compared to whole‐body physiological induction [13, 115]. Efficient inlet coupling and controlled hydraulic resistance are required to ensure reliable capture and transport of sweat from confined regions without premature spreading or evaporation loss. These considerations highlight that electrothermal induction benefits from microfluidic architectures tailored for low‐to‐moderate flow regimes, rather than high‐throughput designs developed for exercise‐driven sweating.

Chemical induction strategies instead rely on cholinergic agonists that mimic neural stimulation of eccrine glands. Passive diffusion of such agents from drug‐loaded interfaces can initiate sweat production under resting conditions; however, diffusion‐limited transport across the skin often results in delayed onset and limited control over stimulation strength. The time required for sufficient drug permeation can reduce temporal precision and complicate synchronization with downstream microfluidic handling. To achieve more rapid and controllable activation, iontophoresis has been incorporated to actively drive cholinergic agents across the skin. Electrically assisted transdermal delivery enables faster onset, tunable dosing, and improved coordination between sweat generation and sampling architecture (Figure 7B) [70].

While early integrated platforms demonstrated closed‐loop induction and sensing, sustained utilization of induced sweat required continuous transport and refreshment mechanisms [58, 62, 97, 139, 151, 152, 153]. To fully capitalize on chemically stimulated sweat generation, microfluidic architectures were incorporated to maintain stable flow and enable real‐time electrochemical monitoring following a single induction event (Figure 7C) [154]. Enabling ongoing refreshment instead of discrete collection allows induction‐coupled sampling to move from episodic measurement toward continuous physiological tracking.

However, even with continuous sensing, sweat secretion typically declines after stimulation, and physiological events of interest may not coincide with the initial induction window. To address this temporal mismatch, some systems embed pre‐programmed induction sequences and architectural delay elements that encode sampling intervals directly within the device. Wearable lateral flow assays integrating narrow cellulose channels with BSA‐modified delay valves direct sweat sequentially into spatially separated detection zones (Figure 7D) [155]. In combination with pre‐programmed iontophoretic activation and self‐powered electrochromic timers, these platforms capture distinct sampling windows without requiring continuous electronic oversight.

Beyond pre‐programmed sequencing, more advanced architectures enable dynamic control over induction timing and sampling location. Autonomous wearable systems incorporate electronically regulated iontophoretic activation across spatially separated compartments (Figure 7E) [156]. With on‐demand switching capabilities, the onset and duration of stimulation at each compartment can be actively controlled. Integrated circuitry coordinates induction timing with electrochemical sensing windows, allowing temporally targeted measurements aligned with specific physiological events. The shift from pre‐programmed sequencing to adaptive electronic control strengthens system‐level coupling of induction and transport, and enables programmable, context‐responsive sweat management.

## 5 Conclusion and Outlook

**FIGURE 8:**
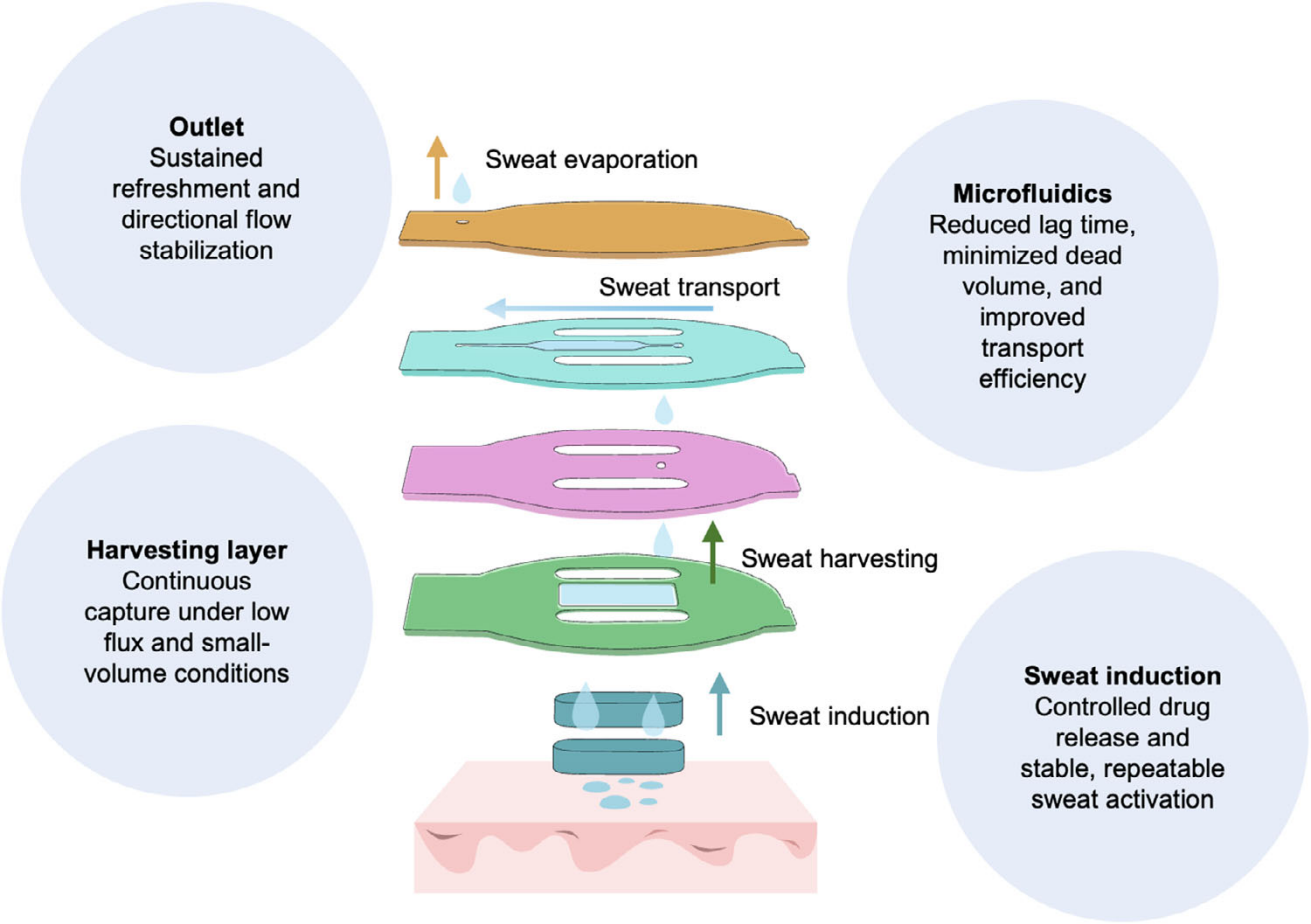
Future design directions for integrated sweat induction and sampling systems. Schematic representation of the multilayer wearable architecture highlighting key engineering challenges across system levels, including controllable sweat induction, continuous harvesting under low secretion rates, microfluidic optimization to reduce dead volume and improve refreshment, and outlet design for sustained directional flow. Addressing these interconnected elements is critical for achieving long‐duration, quantitative sweat monitoring.

As summarized schematically in Figure 8, reliable sweat‐based wearable sensing depends not only on sensing chemistry but on how sweat is induced, intercepted, transported, gated, and refreshed at the device level. Across induction interfaces, inlet design, directional transport, flow regulation, and outlet engineering, mismatches between sweat availability and microfluidic handling capacity frequently limit performance—particularly under low or intermittent secretion conditions. Fluid access, rather than analyte detection alone, often defines the practical boundary of wearable sweat sensing.

Over the past decade, significant advances have been made in individual architectural components, including hydrogel‐based inlets, rectifying microchannels, capillary bursting valves, evaporation‐assisted pumping, and programmable induction platforms. Yet continuous sweat monitoring remains difficult to sustain over extended wear. Sweat secretion is inherently transient, spatially heterogeneous, and strongly dependent on physiological state. Many current systems rely on single or short‐duration stimulation events, limiting long‐term tracking and making measurements sensitive to induction timing.

Looking forward, progress will require moving beyond isolated component optimizations toward fully integrated fluid‐management systems. This includes (i) renewable or programmable induction strategies capable of sustained and repeatable activation, (ii) microfluidic architectures designed to maintain stable refreshment under low flux, and (iii) outlet and sink‐side designs that preserve directional flow over prolonged operation. Importantly, induction modality, fluidic resistance, reservoir volume, and evaporation dynamics must be co‐designed rather than independently optimized.

Standardization will also become increasingly important. Because sweat composition depends on induction context and secretion rate, quantitative interpretation demands clearer reporting of induction conditions, local sweat flux, and refreshment dynamics. These parameters should be defined using quantitative metrics such as local sweat rate, estimated residence time, fluid replacement interval, stimulation duration, and outlet or evaporation boundary conditions. Establishing comparable metrics for sweat availability and fluid replacement will be essential for translating laboratory demonstrations into real‐world deployment. In practical settings, wearable sweat systems must operate under variable conditions, including fluctuations in sweat rate, temperature, and motion. Microfluidic design plays a critical role in maintaining stable sampling under these conditions by enabling controlled transport, minimizing backflow or accumulation, and supporting consistent refreshment.

Beyond hardware, integration with computational modeling and adaptive control strategies may enable closed‐loop regulation of sweat generation and transport in response to physiological feedback. Such systems could dynamically adjust stimulation intensity, flow routing, or sensing frequency based on detected biomarkers, transitioning sweat monitoring from passive measurement toward responsive biofluid management.

Ultimately, making sweat measurable in real‐world settings requires reframing sweat not as a continuously available analyte reservoir, but as a dynamic biofluid whose access must be engineered. By treating induction, sampling, and refreshment as a unified system, future wearable platforms can move toward robust, quantitative, and long‐duration biochemical monitoring suitable for ambulatory and clinical applications.

## Author Contributions

S.S. and W.G. co‐wrote the paper.

## Funding

This work was supported by the National Science Foundation (2145802, 2444815); the Office of Naval Research (N00014‐25‐1‐2258); the U.S. Army Medical Research Acquisition (HT9425‐24‐1‐0249); the Army Research Office (W911NF‐23‐1‐0041); the National Institutes of Health (R01HL155815); the Advanced Research Projects Agency for Health (ARPA‐H‐ICHUB‐24‐101‐504); and the Heritage Medical Research Institute.

## Conflicts of Interest

The authors declare no conflicts of interest.

## Data Availability

The data that support the findings of this study are available from the corresponding author upon reasonable request.
